# Optically Controlled Drug Delivery Through Microscale Brain–Machine Interfaces Using Integrated Upconverting Nanoparticles

**DOI:** 10.3390/s24247987

**Published:** 2024-12-14

**Authors:** Levente Víg, Anita Zátonyi, Bence Csernyus, Ágoston C. Horváth, Márton Bojtár, Péter Kele, Miklós Madarász, Balázs Rózsa, Péter Fürjes, Petra Hermann, Orsolya Hakkel, László Péter, Zoltán Fekete

**Affiliations:** 1 Research Group for Implantable Microsystems, Faculty of Information Technology & Bionics, Pázmány Péter Catholic University, H-1083 Budapest, Hungary; vig.levente@itk.ppke.hu (L.V.);; 2 Chemical Biology Research Group, Institute of Organic Chemistry, HUN-REN Research Centre for Natural Sciences, H-1117 Budapest, Hungary; 3 BrainVisionCenter, H-1094 Budapest, Hungary; mmadarasz@brainvisioncenter.com (M.M.); rozsabal@koki.hu (B.R.); 4 HUN-REN Institute of Experimental Medicine, H-1083 Budapest, Hungary; 5 Two-Photon Measurement Technology Research Group, The Faculty of Information Technology, Pázmány Péter Catholic University, H-1083 Budapest, Hungary; 6 Microsystems Laboratory, Institute of Technical Physics and Materials Science, HUN-REN Centre for Energy Research, H-1121 Budapest, Hungary; furjes.peter@ek-cer.hu (P.F.);; 7 Complex Fluid Research Department, Institute of Solid-State Physics and Optics, HUN-REN Wigner Research Centre for Physics, H-1121 Budapest, Hungary; peter.laszlo@wigner.hun-ren.hu; 8 Sleep Oscillation Research Group, Institute of Cognitive Neuroscience & Psychology, HUN-REN Research Center for Natural Sciences, H-1117 Budapest, Hungary

**Keywords:** brain–machine interfaces, upconverting nanoparticles, drug delivery, biomedical implant, electrocorticography

## Abstract

The aim of this work is to incorporate lanthanide-cored upconversion nanoparticles (UCNP) into the surface of microengineered biomedical implants to create a spatially controlled and optically releasable model drug delivery device in an integrated fashion. Our approach enables silicone-based microelectrocorticography (ECoG) implants holding platinum/iridium recording sites to serve as a stable host of UCNPs. Nanoparticles excitable in the near-infrared (lower energy) regime and emitting visible (higher energy) light are utilized in a study. With the upconverted higher energy photons, we demonstrate the induction of photochemical (cleaving) reactions that enable the local release of specific dyes as a model system near the implant. The modified ECoG electrodes can be implanted in brain tissue to act as an uncaging system that releases small amounts of substance while simultaneously measuring the evoked neural response upon light activation. In this paper, several technological challenges like the surface modification of UCNPs, the immobilization of particles on the implantable platform, and measuring the stability of integrated UCNPs in in vitro and in vivo conditions are addressed in detail. Besides the chemical, mechanical, and optical characterization of the ready-to-use devices, the effect of nanoparticles on the original electrophysiological function is also evaluated. The results confirm that silicone-based brain–machine interfaces can be efficiently complemented with UCNPs to facilitate local model drug release.

## 1. Introduction

In the realm of pharmaceutical technology, the advancement of drug delivery systems (DDSs) stands as a multidisciplinary domain that has garnered significant attention in the 21st century, owing to remarkable strides in materials science and nanomedicine. Nano-drug delivery systems endeavor to surmount the constraints of conventional medicine arising from issues such as inadequate water solubility, limited permeability, extensive dosage demands, and considerable side effects, while also aiming to circumvent direct cytotoxicity and instances of over/underdosing [1].

An extensive amount of research has been conducted in the area of DDSs. Strategies include the use of biomimetic materials (e.g., red blood cell membrane-camouflaged nanoparticles [2,3]), inorganic materials (e.g., silica and iron-oxide nanoparticles [4,5]), hyaluronic acid-based drug nanocarriers [6,7], nanocrystals [8], self-microemulsifying systems [9,10], in situ gels [11], prodrug nanomedicines [12,13], micro electromechanical systems (e.g., microneedles, piezoelectric fibers [14,15]), and microrobots [16]. Various triggers, whether internal (such as pH or enzymatic) or external (like temperature, light, or ultrasound), can activate the ability to deliver drugs precisely to specific sites [17]. Internal triggers are beneficial for singular events, while external triggers provide remote control and noninvasive precision, making them suitable for both singular and multiple events. Among external triggers, light stands out due to its noninvasiveness, high precision, and temporal resolution. However, photoregulated systems face challenges as they typically necessitate high-energy UV or visible light, which has limited tissue penetration and can be carcinogenic. Near-infrared (NIR) light emerges as a promising alternative due to its deeper tissue penetration and safety, but it lacks sufficient energy for triggering photosensitive materials [18].

In response to this challenge in triggering mechanisms, several solutions have been suggested, including two-photon excitation, photoplasmonic systems, Förster resonance energy transfer (FRET), and upconversion (UC) systems. Among these, UC systems, especially those utilizing upconverting/upconversion nanoparticles (UCNPs), have emerged as contenders capable of converting NIR light to UV and visible light. This conversion facilitates the initiation of photoreactions for drug release. By integrating UCNPs with photoresponsive drug carriers, NIR-triggerable drug delivery systems have been developed. Upon NIR laser excitation, UCNPs upconvert incident NIR light to visible light, thus triggering a photoreaction that releases drugs. This methodology has been applied to the in vivo delivery of both small and macromolecular drugs [19,20,21].

UCNPs have the capability to generate UV and visible light in situ, offering two avenues for developing NIR-triggered drug delivery systems. Firstly, this light can instigate alterations in other stimuli-responsive materials, thus changing, e.g., pH or temperature internally [22,23]. Secondly, it can initiate a photoreaction in a photoresponsive compound. Upon light exposure, these photoresponsive molecules undergo chemical changes like photodegradation/cleavage or photoisomerization [24,25]. Drugs can be directly tethered to UCNPs through a photolabile bond, enabling swift release upon irradiation. This approach presents a promising strategy for achieving on-demand drug delivery with precise spatial and temporal control [26].

Light can be used indirectly and directly to mediate drug release by UCNPs externally. For the indirect use of the upconverted light, a combination of gold nanoparticles (NPs) with thermosensitive polymers was employed for the release of encapsulated cargo. Specifically, poly(N-isopropylacrylamide) (PNIPAM) was used in various experiments [27]. For direct use of upconverted light, different strategies were implemented. First, mesoporous nanoparticles were coated with a cis-trans photoisomer. These NPs then underwent gating (physical change) upon light exposure, utilizing the azobenzene photoisomer as the triggerable chemical [24]. Secondly, photodegradable molecule-coated nanoparticles were used. In this approach, gate cleavage occurred upon light exposure, inducing a chemical change. The molecules employed included theo-nitrobenzyl (irreversible change) and spiropyran cinnamates (reversible change) [28]. The third approach was to directly bind the drug to the surface. Here, the drug was bound to the NP surface through a photolabile bond, which cleaved upon irradiation. This method involved o-phosphorylethanolamine conjugated with 5-fluorouracil (5-FU) [29]. The last method that we are aware of is the surface coating of the NPs with polymers. This technique involved the physical or chemical entrapment of a drug in the polymer chains. The polymers used comprise polyethylene glycol (PEG) and polyethyleneimine (PEI) [30].

Nerve tissue is an important target for drug delivery. Neuroscientists are trying to find drug treatments for various diseases and lesions, but an important formula inside the skull, the blood–brain barrier, is a difficult boundary for molecules to cross. Nanoparticles can prove to be a very good alternative to solve this challenge, as with proper synthesis, small particles can be produced that can even passively diffuse through the nerve tissue, via the endothelial cells of the blood–brain barrier (BBB). If this passive diffusion is not enough, different surface modification options can be used to allow the particles to interact with receptors in the brain and then actively (e.g., by receptor-mediated endocytosis) cross the blood–brain barrier [31]. Below, we list some of the 21st century’s most researched diseases in connection with the brain, and some of the ideas where scientists used nanoparticles for possible treatment.

In the context of Alzheimer’s disease, several innovative drug delivery mechanisms have been explored. Piperine-loaded solid lipid nanoparticles (SLNPs) were injected via the abdominal cavity and delivered to the blood–brain barrier via the bloodstream, where they inhibit plaque formation [32]. Polymeric nanoparticles functionalized with tacrine and rivastigmine bind to receptors on brain endothelial cells, facilitating their entry into the brain. Once inside, they enhance learning and memory [33]. The formulation of folic acid-packaged liposomes utilizes passive transport through the lipid bilayer and subsequent degradation [34]. For Parkinson’s disease, a nanoemulsion or SLNPs modified with a dopamine antagonist (rivastigmine) are transported passively and help reduce memory impairment [35]. In cancer therapy, doxorubicin-functionalized gold nanoparticles are designed to enhance the cytotoxicity against glioma and glioma stem cells [36].

Neural response to UCNPs is a key factor when investigating drug delivery mechanisms through the BBB. In several cases, scientists have demonstrated that the biocompatibility of UCNPs is appropriate. For example, in the analysis of both Portioli et al. and Cantarelli et al., UCNPs were cultured with human dendritic cells and significant cell death was not observed [37,38]. Experiments also showed that, under NIR irradiation, even the enhanced growth of neurons along a specific UCNP pattern could be achieved [39]. For the better cellular uptake of the UCNPs, a variety of methods have been developed. One of these is a promising polymer coating, and within it, the PEGylation method [40,41].

In our experiments, from the above-mentioned methods, we used the direct drug (dye) binding and light cleavage mechanism. We applied a complex molecule consisting of a photolabile protecting group (PPG) carrying a quenched fluorophore as cargo through a self-immolative linker. The cargo models a later usable drug. Upon irradiating the UCNP with NIR light, the outcoming visible light can initiate photocleavage of the photolabile bond between the PPG and the linker. Subsequent self-immolation of the linker leads to the release of the fluorophore with reinstated fluorescence. This approach allows for the simple monitoring of the NIR light-induced release process by fluorescence spectroscopy.

To our knowledge, the incorporation of UCNPs to a neural implant surface has not yet been demonstrated in the context of drug or model drug release. There is extensive research on the application of UCNPs on, e.g., polymeric substrates for optogenetics use [42,43], but the model drug delivery capabilities of such a system is still not fully exploited. The described drug delivery system offers several notable advantages. First, if the particles are securely bound to the external interface, the subsequently attached dye or drug will only be released upon exposure to infrared (IR) light. This enables precise temporal control over the release process. Additionally, because the nanoparticles remain immobilized, the spatial release of the nanoparticle-linked chemical can be finely regulated through the application of specific IR light patterns. This allows the targeted molecule to act exclusively on cells in the immediate vicinity of the implant, minimizing off-target effects. Moreover, by modulating the power of the laser irradiation, the amount of the released substance can be adjusted, providing flexibility in dosage. Another critical benefit is the use of IR light, which is less harmful to surrounding tissues compared with other wavelengths. This property is particularly significant in the context of neural applications, where the protection of delicate structures such as neurons is paramount.

In summary, drug delivery via microscale neural implants combined with upconverting nanoparticles, controlled optically through IR light, represents a promising and innovative approach in brain research. This method holds great potential for applications requiring high precision in spatial and temporal drug administration.

## 2. Materials and Methods

### 2.1. Design of the Nanoparticle–Implant Interconnected Layers

**Figure 1:**
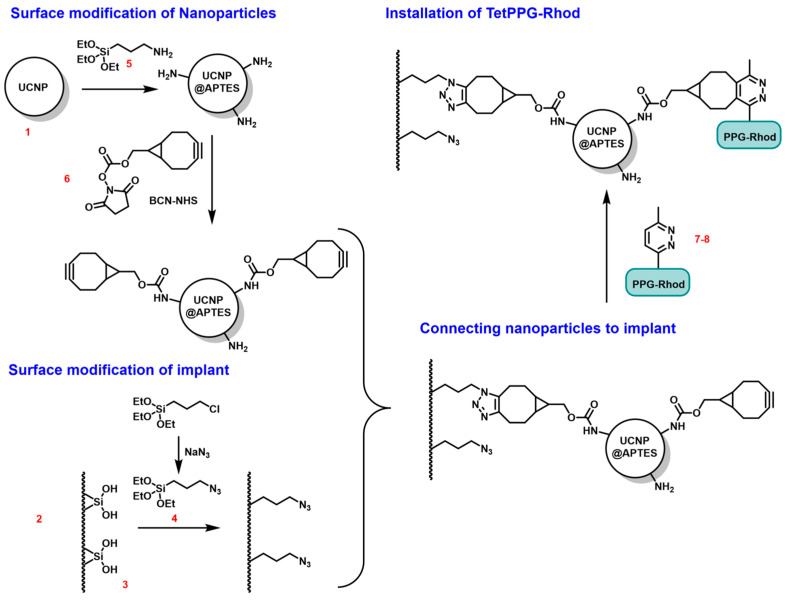
Schematic representation of the surface modification steps.

As shown in Figure 1, the synthesis of the system to be created starts with the modification of the surface of the implant (2) and that of the surface of the upconversion nanoparticle (1), which can be carried out in parallel.

First, an initial layer is deposited on the implant. This involves the immobilization of hydroxyl groups (3) on the surface required for the additional bioconjugation steps, and to achieve the desired hydrophilicity. A possible method is the use of oxygen plasma. The surface of the nanoparticle—in the case of silica coating—already has these OH groups, so its pre-treatment in oxygen plasma is not necessary.

The second step is the polymerization of a silane compound on the surface of both the implant and the nanoparticle. NPTES ((3-azidopropyl)triethoxysilane) (4) with an azide functional group is used on the electrode surface, while APTES ((3-aminopropyl)triethoxysilane) (5) with an amine functional group is used on the nanoparticle.

In the third step, the azide and the amine functional groups, and thus the electrode and the nanoparticles, can be linked by the compound BCN-NHS (bicyclononin-N-hydroxysuccinimide) (6), with two side chains that have different functions. The BCN side can react with both the azide group on the implant surface and the tetrazine ring of the dye, while the ester side of NHS can react with an amine group of the nanoparticle.

**Figure 2:**
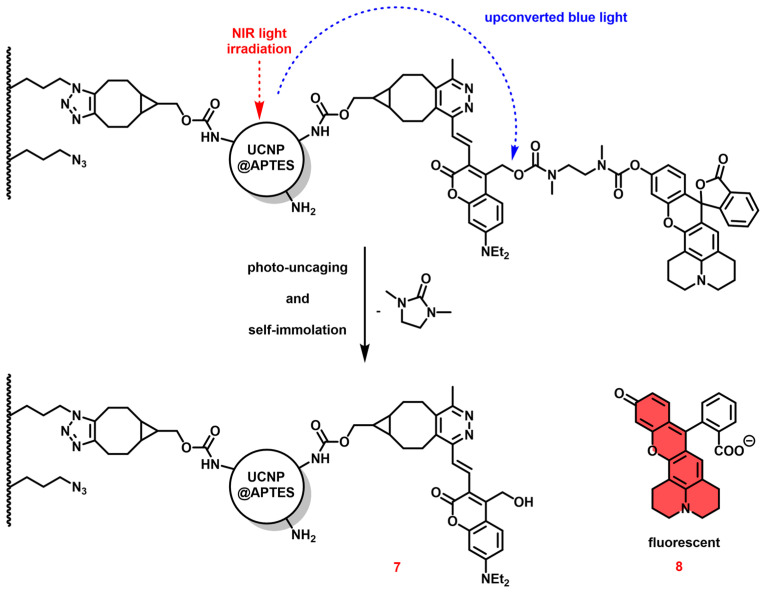
Uncaging (photocleavage) mechanism after NIR light exposure of the UCNPs.

To illustrate the application in vitro and in vivo, a special PPG-cargo construct (7–8), abbreviated to the name of TetPPG-Rhod (vinyl tetrazine-coumarin photolabile protecting group-bound rhodol), is linked to the system. Half of the BCN-NHSs is located on the opposite side of the nanoparticle to the implant (i.e., not reacting with the azide groups on the model surface), thus the tetrazine group of the dye is able to react with the triple bond of the BCN ring. Due to the fluorogenic nature of the TetPPG, its fluorescence is turned on upon reaction with BCN, allowing for confirmation of covalent attachment of the construct to the surface. A further unique feature of TetPPG is that it only releases its cargo (Rhod) upon blue-light (450 nm) irradiation, once it is attached to the particle through a reaction with BCN. By using UCNPs that upconvert the light to this wavelength range, the rhodol molecule (8) can be detached from the surface in response to NIR excitation of the nanoparticle. This process is called uncaging, as illustrated in Figure 2. After the NIR irradiation, the fluorescent signal of the rhodol can be detected.

### 2.2. Materials and Fabrication

We modified the surface of an ECoG (electrocorticography) electrode array called Micro 8 Hexagonal °AirRay Grid Electrode (Figure S1a), which was purchased from CorTec GmbH (Freiburg, Germany).

The recording sites of the ECoG were made of a platinum–iridium (Pt–Ir) alloy, with 0.3 mm diameter recording sites in a hexagonal contact arrangement. Platinum possesses low concomitant impedance, high charge transfer capacity, and high electrochemical stability, while the mechanical hardness and stiffness of iridium provide high bend resistance for the electrode as well.

The substrate that hosts the recording sites was made of silicone rubber. This material is a widely utilized siloxane polymer, with the chemical formula of [R_2_SiO]_n_ where R indicates the functional groups that are attached to the silicon atoms (e.g., methyl groups in PDMS). Unique properties of this chemical are its high flexibility (1–5 kPa) and stretchability elongation (up to 600%), optical transparency, nontoxicity, biocompatibility (USP Class VI), low thermal conductivity, and long-term thermal stability. These traits made it applicable as an insulator for the recording sites in our electromechanical device.

In some of our preliminary experiments, we utilized only PDMS disk cut-outs instead of the silicone rubber-based ECoGs. For this purpose, we used the standard Sylgard 184 elastomer kit (Dow Corning Corp., Midland, MI, USA). The mold was prepared by mixing 10 parts of base with 1 part of curing agent, then we vacuumed out the air bubbles, baked it, and at the end, cut out 0.5 cm diameter disks from it.

Parallelly, as mentioned in the Introduction, we also altered the surface of the NaYF_4_:Yb/Tm-based UCNPs, with APTES and BCN-NHS. The water-dispersible UCNPs were not synthesized by our research group but made by the company Enlipsium, Singapore. These 50 nm size silica-coated nanoparticles have a NaYF_4_:Yb/Tm matrix-core and NaYF_4_ shell (Figure S2). Their excitation and emission wavelengths are 980 and 475 nm, respectively.

#### 2.2.1. Plasma Treatment

Plasma treatment is used to activate the electrode surface by creating silanol terminations (Si-O-H) on it, while also removing any impurity that is on the substrate beforehand. The plasma treatment was performed in a closed vacuum chamber with a low-pressure plasma system (Pico, Diener Electronic, Ebhausen, Germany). While optimizing the experimental parameters, we were able to induce the highest hydrophilicity on the silicone surface by applying 50 Watt power for 50 s-long reaction time, so we used these settings later on. Additional settings of the Pico device were as follows: 3 min pumping down time on 0.30 mbar pressure with O_2_ and air process gases, 2 min of O_2_ gas supply with 100% gas share and 30 sccm gas flow (15 min, 45 max) on 0.5 mbar (−0.2 mbar and +1 mbar maximum deviation), pulsed plasma power mode (4% maximum absolute power deviation) with 47% C-Load and 48% C-Tune, 10 s flushing time, 10 s venting time.

The effect of the plasma treatment lasted for about 30 min; therefore, as time passed, the hydroxyl groups became more unstable and were more easily replaced. Consequently, the following modification was carried out within this time interval.

#### 2.2.2. NPTES/APTES Modification

The ethyl groups of organic trialkoxy silanes condense with the OH groups on the surface of the nanoparticle or the electrodes and can form stable siloxane (Si-O-Si) bonds while ethanol leaves the system. They can also form these siloxane bonds between themselves, i.e., self-condensing, forming gel-like, oily oligomers. Due to the much lower density of these unwanted by-products, they can be removed from the solutions by repeated centrifugation and then redispersion steps. APTES (and to a substantial extent NPTES) is readily soluble in ethanol, forming a stable solution at its natural pH, which leads to rapid condensation.

The general steps for modifying nanoparticles with APTES are as follows: First, a 50 µL, 1 mg/mL suspension of nanoparticles and 75 µL of Dimethyl sulfoxide (DMSO, Merck KGaA, Darmstadt, Germany) are mixed in an Eppendorf pipette. Then, repeated ultrasonication and shaking are used to separate any aggregated particles. Into each Eppendorf tube, 15 µL of concentrated (100%) APTES (Merck KGaA, Darmstadt, Germany) is added dropwise. This is performed in a glove box under a nitrogen atmosphere to avoid contact of the APTES with oxygen and thus to avert self-condensation. After another cycle of ultrasonication, the substances are allowed to react overnight while being shaken at 150 1/min at 22 °C. The next day the solution is vortexed at 1500 1/min for five minutes, and after that, a three-step cleaning process is repeated 3 times (step 1. centrifugation for 5 min at 14,500 rpm, step 2. supernatant is pipetted out and 0.1 mL of fresh DMSO is added back, step 3. ultrasonication for 5 min).

In the meantime, the steps to modify the electrode surface with NPTES were carried out as follows. We prepared the NPTES solutions by mixing 80 µL NPTES with 320 µL DMSO. Then in 1-1 screw-top vial (later sealed with Parafilm), the mixture was shaken to prevent evaporation. The electrodes were placed separately in the vials to prevent clumping. The samples are vortexed at 500 rpm for 1 h. For the final step, we let the reaction take place overnight.

#### 2.2.3. BCH-NHS Alteration

BCN-NHS is a heterobifunctional compound, i.e., it is composed of two subunits with different functions. The BCN side can be used to perform the so-called click chemistry azide-alkyne cycloaddition, while the other side is an NHS-activated ester that can hydrolyze. The BCN-NHS is suitable for aqueous-based bioconjugations because of its hydrophilicity and its reactivity with azide and amino groups.

The modification steps of the nanoparticles BCN-NHS were as follows. Solutions of 3.6 mg of BCN-NHS (Merck KGaA, Darmstadt, Germany) and 50 µL DMSO were prepared. To obtain the correct basic character of the solution (pH of about 8), 1 µL triethylamine was added to the solution. To the BCN-NHS solution, the 100 µL of APTES-modified nanoparticle dispersion was added, then vortexed at 1500 1/min for 1 h.

#### 2.2.4. Connecting the Nanoparticle to the Surface

The reaction between BCNs and the azide groups was a crucial step in the whole process flow, so we tested it various times. We used different reaction times for this measurement, but a rather long, one-week duration was found to be ideal.

The connection steps were as follows. Cleaning (with centrifugation and ultrasonication) of the BCN-nanoparticle solution was repeated 3 times (at the final cycle, before the sonication, 400 µL of fresh DMSO was added back instead of 100 µL). The electrodes were washed with ethanol and dried with N_2_ gas. The electrodes were placed in the Eppendorfs containing the NP solution. Because the reaction between BCN and the azide groups was relatively slow, we let it be completed over one week.

#### 2.2.5. Usage of the TetPPG-Rhod Construct

The specific construct that we used was abbreviated as TetPPG-Rhod. This compound is based on a rhodol molecule bound to a vinyl-1,2,4,5-tetrazine-coumarin photolabile protecting group (PPG) through a self-immolative linker.

Tetrazine can react with strained cycloalkynes (in this case a bicyclononyne, BCN) containing double or triple bonds, under physiological conditions, with the emission of dinitrogen. This is one type of click reaction and a bioorthogonal reaction at the same time, allowing fast and efficient covalent surface modification of nanoparticles.

To install TetPPG-Rhod onto BCN-functionalized UCNP surfaces, 0.4 mL of a 0.01 mg/mL TetPPG-Rhod–dimethylformamide suspension was added to an Eppendorf and shaken for 5 min. After that, the nanoparticle-modified electrodes were soaked in the TetPPG-Rhod solution. The system was allowed to react overnight while being covered with aluminum foil. Before the fluorescence measurements, the dye-modified electrodes were soaked in ethanol to get rid of the excess dye that was not chemically bound to the particles.

**Figure 3:**
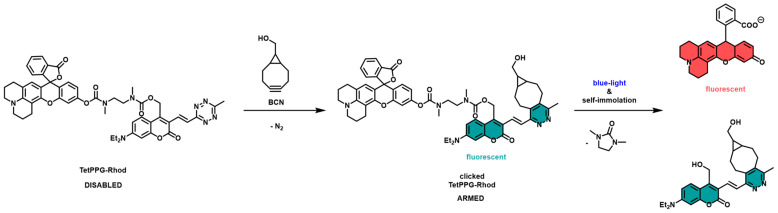
The molecular structure of TetPPG-Rhod, the click and the uncaging process [44]. NMR spectrum of the dye system can be found in the Supporting Information of the cited article.

Upon irradiating the UCNPs with NIR light, the outcoming visible (450 nm, blue) light is expected to result in photocleavage leading to the release of the rhodol moiety. When rhodol becomes free, its emission can be detected under a fluorescent microscope, while without the photocleavage step, the rhodol emission is virtually zero. This is the mechanism called uncaging (see Figure 2 and Figure 3).

### 2.3. Measurement Setups

#### 2.3.1. Contact Angle

The contact angle (θ) is a qualitative measure of the wetting of a solid by a liquid. In geometric terms, it is the angle formed by a liquid at the three-phase boundary between the gas and the solid at the interface [45]. When preparing the silicone surface, the contact angle provides useful feedback on how the modification of the sample affects its hydrophilicity, i.e., whether the functional group (e.g., hydroxyl or amine) under investigation has been transferred to the surface. As a model electrode surface, we used PDMS disks which were modified the same way as the silicone ECoG arrays.

For the contact angle measurements, we used a syringe capable of making 1 µL distilled water droplets, while utilizing a high-powered LED to shine a light through the surface of the droplets and then took a picture of them with a CCD camera on the opposite side. The resulting images were analyzed using a software called ImageJ 1.54 and its Contact Angle plugin.

#### 2.3.2. FTIR

The Fourier Transform Infrared Spectroscopy/FTIR measurements were performed with a Bruker (Billerica, MA, USA) TENSOR II spectrometer applied with a liquid nitrogen cooled, MCT/mercury cadmium telluride detector and a Specac (Orpington, UK) Golden Gate ATR (Attenuated Total Reflectance) sensor crystal. We used the associated OPUS 7.0 software package for data evaluation.

Both in the case of the contact angle and the FTIR setups, samples made from PDMS disks were modified up to each chemical step and then measured to see whether the attachment of these chemicals gave a signal regarding the disks’ hydrophobicity and their transmittance.

#### 2.3.3. SEM

Scanning Electron Microscopy/SEM measurements were carried out with a TESCAN (Brno, Czech Republic) MIRA3 system with the detection of either the secondary electrons or those and the backscattered electrons together. After being placed in the vacuum chamber, the UCNP-modified ECoG arrays were examined at magnifications ranging from 4 to 100 thousand times (working distance and detector type varied in some cases) with the acceleration voltage of 1 keV.

To make SEM recordings more quantitative, the images were analyzed using MATLAB (Mathworks Inc., Natick, MA, USA) R2024a. On the greyscale converted images, a Gaussian filter was applied first, to remove unwanted noise. After that a Canny-type edge detection was performed, followed by a binarization based on the appropriate threshold. Subsequently, an opening and a closing morphological operator was applied to the images, after which, markers were also prepared for the watershed segmentation. Based on the markers, the program was able to separate the individual objects (particles and their groups) and number them. Based on all this, as a final step, it was possible to calculate what percentage of the image’s pixels were covered by the segmented objects.

This setup was used for various reasons. The most important one was to observe how the particle layers formed due to different modification parameters and to examine what the particle distributions were on different substrate regions, but determining the actual size of the UCNPs was another relevant reason. While iterating through different modification parameters, the created samples were measured with SEM.

#### 2.3.4. EIS

For the electrochemical impedance spectroscopy (EIS) measurements, a Gamry Reference 600+ system (Warminster, PA, USA) was used, which is a high-performance potentiostat/galvanostat designed for precise, low-current measurements. Using the Gamry hardware and the Gamry Framework 5.6 software, the impedances of the ECoG sites could be measured only one by one but with high accuracy. The working, reference, and counter electrode systems were soaked in a phosphate-buffered saline (PBS) solution with a concentration of 0.01 mol during measurement. This solution served as the electrolyte solution. A silver/silver chloride electrode (Ag/AgCl, 3 M KCl, Bioanalytical Systems, Inc., West Lafayette, Indiana, USA) with a flexible connector was used as a reference electrode. In all cases, the measurement was carried out in a Faraday cage. Before each measurement, the ECoGs were washed with ethanol and then connected to the potentiostat.

In the EIS setup, 4 unmodified and 4 UCNP-modified ECoG were tested to determine how the particle deposition has affected the average impedance response of the exposed electrode surface.

#### 2.3.5. Fluorescence Spectroscopy

For carrying out the fluorescence spectroscopy measurements, a Jasco FP-8300 spectrofluorometer (Jasco UK Ltd., Heckmondwike, United Kingdom) was used, along with the Spectra Manager 2.5 software). Excitation was set to 535 nm, while detection was in the 550–700 nm range. The device was in high sensitivity mode, with a bandwidth of 5 nm-s and data intervals of 1 nm. Excitation of the UCNPs was achieved with an infrared laser emitting in the 980 nm wavelength range. The threshold current of the laser was found to be 71 mA, and the focal point of the outgoing light was 9.43 mm from the plane of the metallic end of the device.

Two fully modified ECoG devices were used in this setup, one to observe the uncaging reaction in general after various periods of infrared light exposure, and another to observe the reliability of the system, i.e., what effect does natural light have on the device (on its uncaging feature). In the latter case, starting from the acquisition of the dye, we performed the experiment in a dark environment during the functionalization, and then we wrapped the Eppendorf in aluminum foil after each step. When it was time to measure the fluorescence, we removed the foil surrounding the samples and measured the amount of dye that could be washed off the surface in the dark. Then we placed the electrode next to the window for 60 s, allowing the sun to shine on it, recorded the spectrum of the dye supernatant, and repeated this with 240 s of natural light. After that, we performed 60, 120, and 240 s IR excitation—fluorescence measurement cycles.

Since we are not yet working with real drug molecules, in both cases, uncaging (model drug release) efficiency could be proved by detecting the dye’s rising fluorescence in the supernatant.

#### 2.3.6. Two-Photon Microscopy

Two-photon microscopy is a prominent method to measure and modulate cellular activity deep in the live brain with high spatial resolution and activate photolabile compounds for localized intervention.

In vitro two-photon microscopy was performed on a fully functionalized ECoG system (and an unmodified PDMS disk) using a FemtoSMART-Dual microscope (Femtonics Ltd, Budapest, Hungary), equipped with a 20× 1.0 NA objective (XLUMPLFLN, Olympus Corp., Tokyo, Japan), a tunable high-power Ti:Sapphire laser (Chameleon Discovery Ultra II, Coherent Corp., Saxonburg, PA, USA) tuned to 980 nm, a H11706P-40 GaAsP photomultiplier tube (Hamamatsu Photonics, Shizuoka, Japan), and an ET520/60m-2p dichroic mirror (Chroma ATE Inc., Taiwan). Measurements were recorded with and analyzed in MES (Femtonics Ltd., Budapest, Hungary) and MATLAB (Mathworks Inc.).

#### 2.3.7. Electrophysiology

All experiments were performed in accordance with the guidelines and approval of the Animal Care and Experimentation Committee of the Institute of Experimental Medicine. All procedures complied with Hungarian and European regulations for animal research. One wild-type C57Bl/6J mouse (110 days old) was used. The mouse was sourced from the animal facility of the HUN-REN Institute of Experimental Medicine. Animals were housed in a temperature-controlled environment (24 ± 1 °C) on a 12 h reverse light cycle (dark period between 08:00 and 20:00) and humidity between 40 and 70% and were kept in small groups (2–4 mice/homecage) in enriched environment with cardboard rolls, rotary discs, and extra nesting material (sizzle pet). The mice had ad libitum access to food and water.

For the acute in vivo measurement, the mouse was anesthetized deeply with a mixture of ketamine (90 mg/kg)—xylazine (10 mg/kg). Additional doses were administered regularly to prolong anesthesia. Ropivacaine (Astra-Zeneca, 0.2%, 0.05 mL) was applied subcutaneously over the skull prior to the surgery. An ophthalmic ointment was applied to the eyes to prevent drying during surgery. The mouse was kept warm throughout the surgery and subsequent acute experiment with a heating pad (Supertech TMP-5b-SFAC-5). The skin on the skull was removed and a reference electrode was implanted over the left cerebellum, fixed with a mixture of instant-curing glue and dental cement. After curing, a craniotomy accommodating the whole device was made over the left hemisphere with a 0.3 mm burr drill bit. The *dura* was not removed. The UCNP-ECoG device was laid over the cortex and covered with a custom rectangular cover glass, which was bonded to the skull with the same glue mixture.

The mouse was then moved to the recording setup. Multichannel electrophysiology and recording site impedance were recorded using an RHD2132 amplifier board (Intan Technologies LLC., Losa Angeles, CA, USA) with an amplifier bandwidth from 0.1 Hz to 7.5 kHz, sampled at 20 kHz. After the measurements, the mouse was terminated with cervical dislocation, and the device was recovered.

## 3. Results and Discussion

### 3.1. Contact Angle Measurement

**Figure 4:**
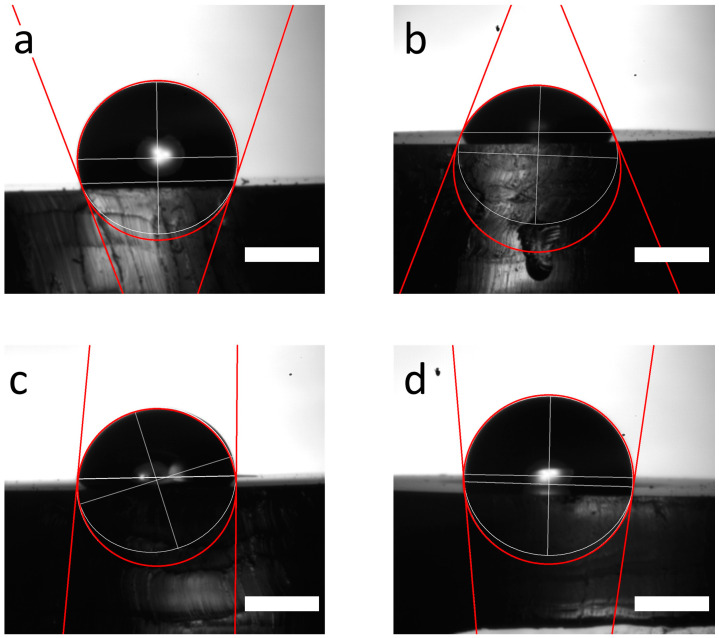
Contact angle of the modified silicone (PDMS) model substrate. (**a**) Bare substrate, (**b**) plasma treated substrate, (**c**) NPTES-treated substrate, (**d**) BCN-NHS-modified substrate (scalebars indicate 1 mm).

As illustrated by Figure 4., there was a significant difference in the θ angles between each modification step. In the beginning, the contact angle was found to be high, 107.4° (the white V shape is open at the top half of Figure 4a), because PDMS (or any other silicone for that matter) as a bare material is highly hydrophobic [46]. Plasma treatment created OH terminals on the surface, making it more hydrophilic, though decreasing θ all the way down to 63.3° (the white V shape is open at the bottom half of Figure 4b). Because azide and NHS groups are not as hydrophilic as OH groups, we expected an increase in the contact angle again when these chemicals were introduced to the surface. As expected, the attachment of azide raised the contact angle to 80.3°, while the NHS groups raised it even higher, to 91.5° (Figure 4c,d).

### 3.2. FTIR Measurement

Similarly to the contact angle measurement, we also investigated how the IR spectra of the modified surfaces, i.e., the relative absorption (RAE/relative absorbance unit) for a given wavenumber, changed during the bonding process with the compounds used. For the evaluation, we used several reference absorption tables that indicated the intensity peaks that in principle belong to each functional group.

The initial spectrum of the PDMS is shown first, while for the modifications, the spectrum from which the background values have been subtracted can be seen. This is necessary because, in many cases, the change in the spectrum is so small that it can only be properly visualized this way.

**Figure 5:**
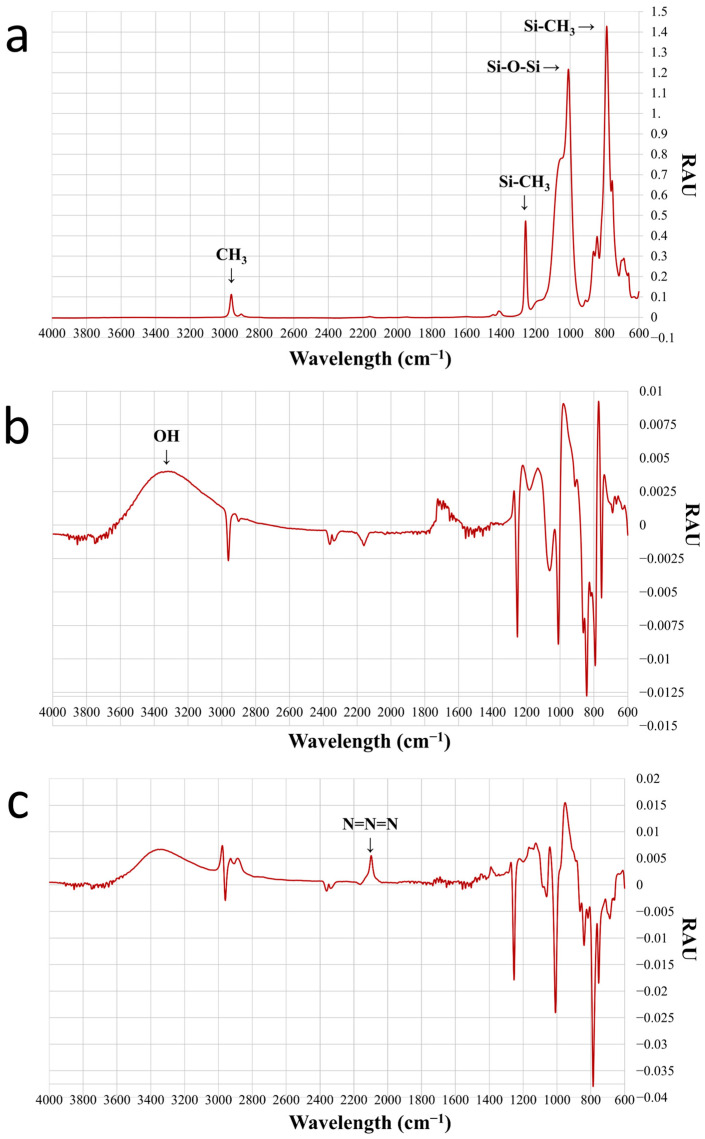
FTIR spectra recorded after each modification step of a PDMS disk. (**a**) Bare substrate, (**b**) plasma-treated substrate, (**c**) NPTES-treated substrate.

By default, the C-H bonds of the methyl groups in PDMS gave a signal at about 3000 cm^−1^. The binding of 1-1 methyl groups to a Si atom was measured in the 800 and 1300 cm^−1^ regions, while the two Si atoms surrounding an oxygen caused a strong increase in absorbance at 1000–1100 cm^−1^, as shown in Figure 5a. The surface coverage of the hydroxyl groups was confirmed by two observations: Firstly, the increase in relative absorbance in Figure 5b between 3100 and 3600 cm^−1^ wavenumbers, which is characteristic of oxygen-hydrogen bonding [47]. Secondly, the decrease in the peak around 3000 cm^−1^ is typical of the C-H bonding of methyl groups, and the complete disappearance of the peak around 1300 cm^−1^, is distinctive of the bonding of carbon to silicon (reduced below 0 due to backgrounding). After the NPTES molecules were able to bind to OH groups, a spectral change could be detected at 2100 cm^−1^ which is distinguishing of the azide fragment. The absorbance increased to 0.005 in this case (Figure 5c). At the last step, the disappearance of this 2100 cm^−1^ azide peak indicated that the azide-alkyne cycloaddition had taken place, i.e., the BCN-NHS formed the outermost layer.

### 3.3. SEM Measurement

**Figure 6:**
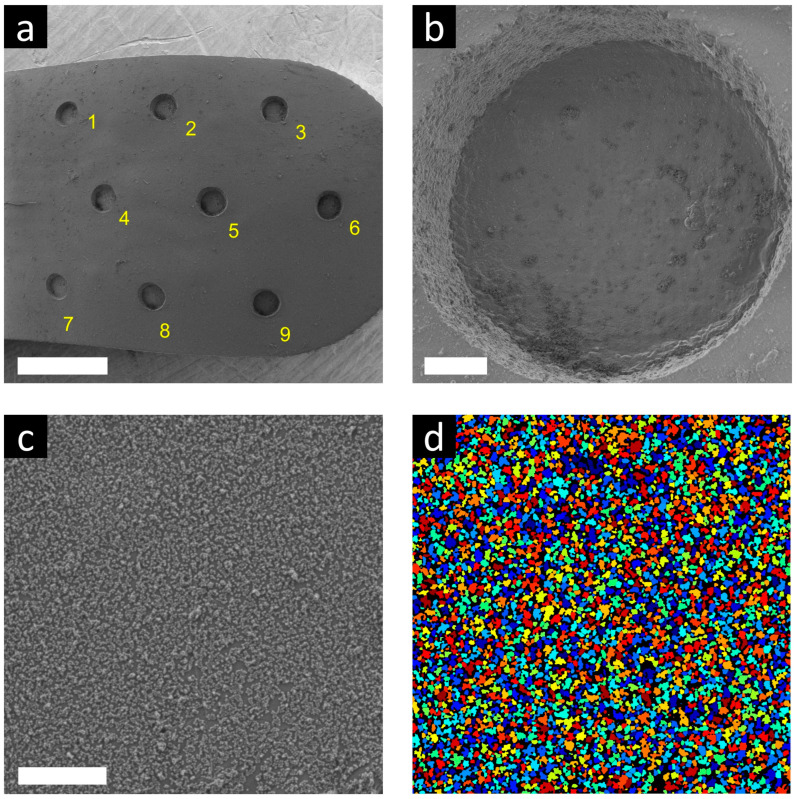
SEM images of the UCNP-ECoG system. (**a**) The whole probe surface with numbered recording sites, (**b**) a closer look at recording site 9 at larger magnification, (**c**) UCNP coverage on the substrate surface near site 4, (**d**) particles detected with Matlab, near site 4 (white scalebars show 1 mm, 50 µm and 2 µm, respectively).

To simulate the mechanical effect of implantation into the nerve tissue in vitro, we used a so-called agar gel test [48]. With this very simple method, we were able to determine whether the particles were still attached to the surfaces after a physical impact like the conditions during implantation. In total, 300 mg of agar powder with 20 mL of distilled water were mixed. This solution was heated to boiling point with continuous magnetic stirring and then it was poured into a two-well plate. The cooled, solidified agar gel became similar in consistency to nerve tissue. The UCNP-functionalized silicone ECoG was placed on top of the gel and gently pressed onto it several times. Then, any detached nanoparticle was washed off with ethanol and the remaining agar gel. After drying them with nitrogen gas, they were taken for microscopic examination. Figure 6 shows SEM images of one of these tested ECoGs.

Higher magnification SEM images were also taken of several substrate regions of the ECoG electrodes near their measurement points (labeled 1–9). Using a custom Matlab code, image analysis was also performed on the regions to detect the attached nanoparticles. The result was primarily a surface coverage percentage and secondarily a detected object number (individual particles and aggregated particle groups summed). Table 1 shows the calculated parameters of each region on three devices and, in the last column, the average of the regions. The substrate regions had 52.40% coverage, with 5047 detected objects on average. Region 2 had the lowest of both measures. It is worth observing Figure 6c,d where we show the original SEM image of the best-covered area, namely device 2, region 4, and the detected objects on it, marked with randomized colors.

### 3.4. EIS Measurement

The EIS measurements were used to indirectly prove that the attachment of the nanoparticles does not change the impedance of the device to such an extent that electrophysiological measurements cannot be performed with it later (i.e., UCNP presence caused by physical absorption to the sites is negligible in view of the impedances).

The frequency range of EIS measurements is usually set to an interval between 1 and 10,000 Hz, which is commonly used in neuroscience. This is practical because individual neurons (or groups of neurons) give a measurable signal (firing) in this range. Local field potential signals containing information on slow synaptic potentials range from 1 to 1000 Hz, while single/multiunit activity is typically recorded at higher frequencies between 1000 Hz and 10 kHz [49].

**Figure 7:**
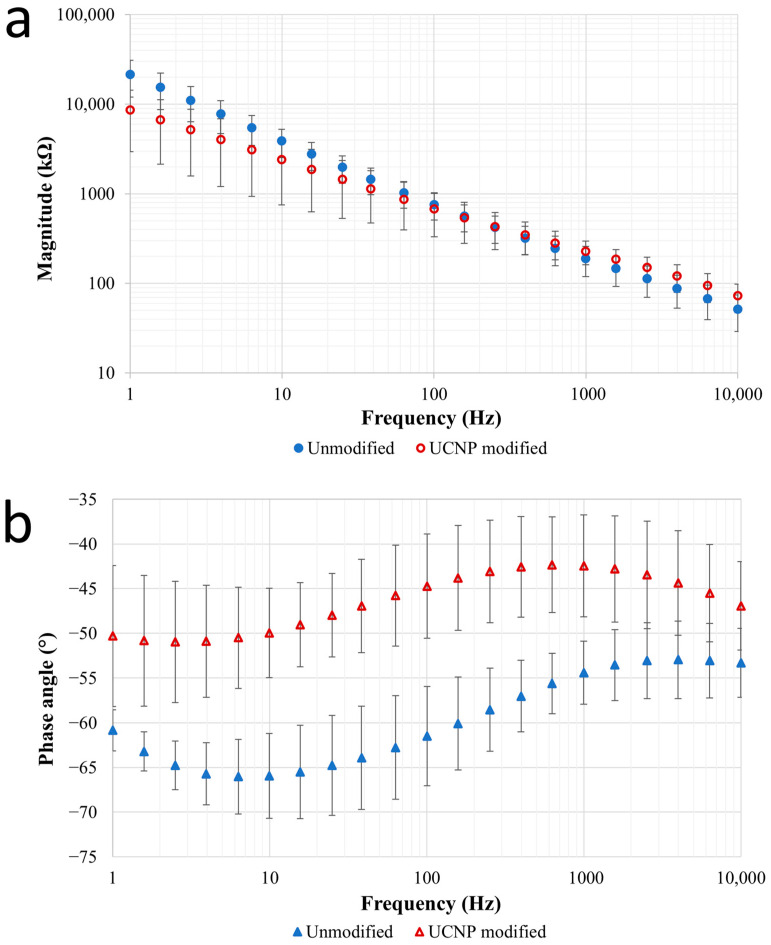
EIS measurements of the ECoG systems, represented on Bode plots. (**a**) Resistance (magnitude) of the unmodified and UCNP-modified ECoGs, (**b**) reactance (phase angle) of the unmodified and UCNP-modified ECoGs.

Figure 7a,b show the Bode plot characterizing the recording sites of four ECoG arrays hosting UCNPs compared with the unmodified control. At 1 kHz, the average absolute value of the site impedances changed from 189.53 kΩ to 229.24 kΩ. This slight increase is not expected to influence the behavior of the recording arrays, as the typical impedance of ECoG recording sites of similar surface areas at 1 kHz is also in the same regime [50,51].

For the phase angle, it was observed that the course of the curves was nearly the same before and after the modification, and no opposite phase angles were obtained, which leads us to conclude that the circuit elements of our device behave in a similar way in terms of their operation.

### 3.5. Fluorescence Spectroscopy Measurement

During our fluorometric measurements, we investigated whether the TetPPG-Rhod conjugate was properly fixed on the nanoparticle surface. It is important to note that only BCN-reacted conjugates could undergo the photo-uncaging step, while free, unreacted conjugates could not.

**Figure 8:**
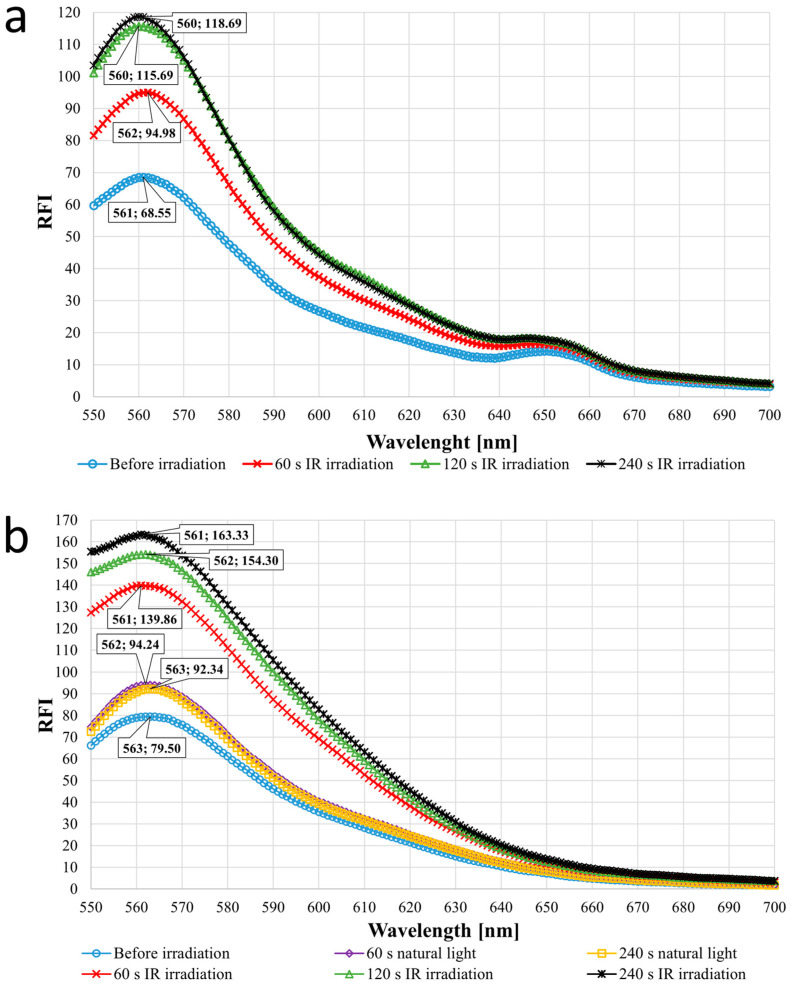
Fluorescence spectroscopy measurements of the ECoG systems. (**a**) RFI (relative fluorescent intensity) changes due to the IR laser irradiation, (**b**) Observing the effect of natural light vs. IR laser light on the system.

As shown in Figure 8a, the emission after 60 s of NIR excitation was the lowest, with an RFI (relative fluorescent intensity) of exactly 94.98. Then, after excitation for 120 s, the dye signal increased to 115.69 and to 118.69 after 240 s. Between the peaks of the wave marked in blue (before IR) and red (after 60 s IR) on the graph, there was a 1.39-fold increase, while between red and green (after 120 s IR), the fold was 1.22. Between the two longest irradiation times, the increase was only 2.6%. Comparing the longest excitation and the one without irradiation, the increase was 73.14%. This measurement was encouraging as we demonstrated that the UCNP system not only works in solution but is also immobilized on an electrode. Thus, the particles were able to bind in sufficient amounts to the substrate regions of the electrode and then, after IR excitation, they could also convert the IR light into blue light even in this surface-bound state [27].

To complete the fluorescence spectroscopy measurements, we tested how sensitive the system (specifically the dye) is to natural light, i.e., whether the non-specific uncaging of rhodol is occurring even without NIR irradiation. The results of this type of experiment are represented in Figure 8b. Before the sample was exposed to natural or NIR light, an increase in fluorescence intensity up to 79.42 RFI was observed. This is probably because the dye is not only able to bind to the surface of UCNPs but also to diffuse into the PDMS substrate and then escape (this is also the case in the previous measurement). Later on, this phenomenon can be eliminated by thoroughly soaking and washing the substrate material several times. Under the influence of natural light, the fluorescence intensity could not increase higher than 94.24 RFI even after 60 and 240 s of irradiation, so the increase was 18.66% compared with the case without irradiation. When the NIR laser was applied, the RFI increased with irradiation duration. The RFI was 139.86 at 60 s, 154.3 at 120 s, and 163.33 at 240 s of irradiation, so the increment is 48.4%, even with the shortest irradiation time. Between the shortest and longest laser illumination time, the increase in RFI was 16.78%. To summarize, the substrate material’s physical composition allows the system to deliver a minor amount of dye without external influence, but the blue wavelength range of natural light can only cause dye molecule cleavage to a small extent. The much higher maximum intensity, of one and a half times the case of natural light, can only be achieved with particle-converted light after laser excitation, thus proving the system to be reliable.

### 3.6. Two Photon Uncaging Test

**Figure 9:**
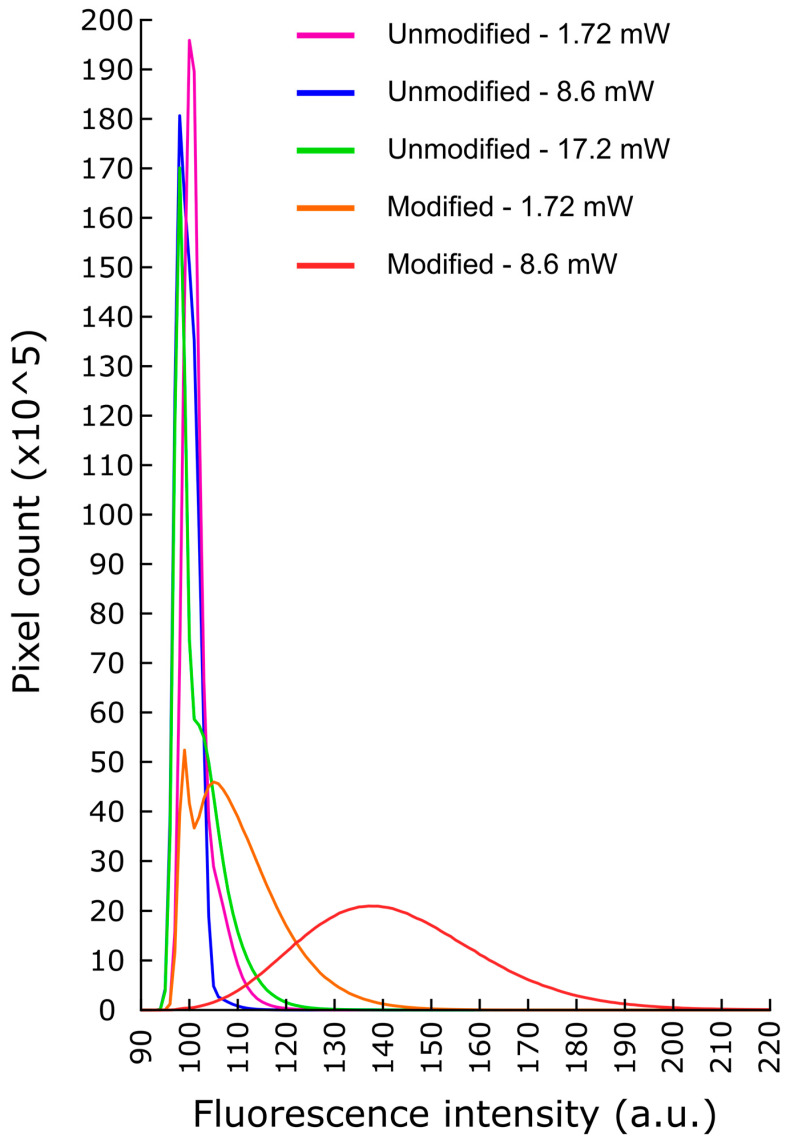
Averages of the image histograms of 2P images taken on sample regions of unmodified PDMS discs and a fully modified UCNP-ECoG device with different light intensities. The histograms of UCNP devices (orange, 1.72 mW; red, 8.6 mW) show increased fluorescence on these images, suggesting successful uncaging.

Two-photon uncaging tests were performed in vitro to validate the uncaging capability of the ECoG device. Unmodified PDMS discs and fully modified devices were tested to compare the fluorescence change caused by two-photon excitation. The devices were placed under the objective, using distilled water as immersion, with the modified surface facing up towards the incoming excitation light. Several regions of interest (200 µm by 200 µm) were scanned pixel by pixel (0.22 µm/pixel, 0.0198 µs pixel dwell time for every image), uncaging the dye molecules while taking an image. The resulting images served as samples to measure the fluorescence increase due to the uncaged dye molecules. Histograms of these images, showing the discrete values of fluorescence units (arbitrary units, a.u.) and the number of pixels (counts) on the image with that value, were calculated and averaged. The detector of the microscope was configured to the baseline value of 100 a.u. with minimal deviations around this value due to voltage fluctuations; therefore, images taken without illumination would have most of the pixels distributed close to this baseline value. Figure 9 shows the averaged histograms of unmodified PDMS discs and fully modified devices according to the light intensity used for imaging (1.72 mW, 8.6 mW, 17.2 mW). Two-photon imaging (and by the experimental setup, also uncaging) of regions of the unmodified PDMS discs with increasing light intensity (1.72, 8.6, and 17.2 mW, 23, 13, and 10 separate regions, respectively) all produced a distribution centered on the baseline value of 100 a.u. (98, 100, and 98 arbitrary units of fluorescence on average, respectively), suggesting that fluorescence was below detection. However, when two-photon imaging was performed on fully modified devices, notable shifts of the averaged pixel distributions could be observed on the images. Imaging the fully modified devices with 1.72 mW (8 regions) produced a second peak on the histogram (mean = 105 a.u.), while imaging with 8.6 mW (6 regions) eliminated the baseline peak and resulted in a single Gaussian distribution with a mean of 137 a.u. This indicates that fluorescence was detected during the imaging, resulting in more pixels with values higher than baseline. The increased fluorescence of the modified device suggests that two-photon illumination was able to cause the release of dye molecules from UCNPs on the surface, confirming the functionality of the uncaging scheme.

Imaging the modified devices with 17.2 mW with the described settings evoked bright artifacts that were not observed on unmodified PDMS samples, which prevented comparison at this and higher light intensities. Nevertheless, further experiments need to be carried out to determine the quantity of the dye/drug molecules releasable at specific illumination parameters.

Model dye release was not yet demonstrated on the brain surface, only under in vitro circumstances. At this current phase, our interest was only to prove that the UCNPs emit enough photons that release the dye molecule. Applying neurotransmitters that are capable of the uncaging mechanism and delivering them from the implant surface would be our final goal, but it was not in the scope of this research. Releasing these neurologically active chemicals from the nanoparticle surface would result in a change in brain activity which could be measured through electrophysiology.

### 3.7. Electrophysiology Measurement

**Figure 10:**
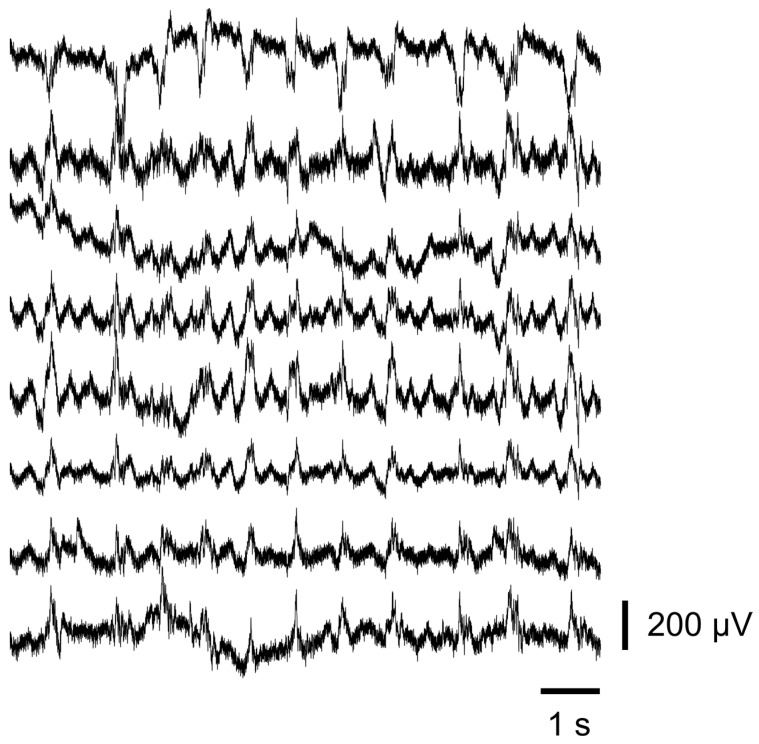
Sample in vivo electrophysiological recording with a UCNP-modified ECoG device from a ketamine–xylazine anesthetized mouse, showing characteristic oscillations evoked by the anesthesia. The 8 traces are simultaneous recordings from the 8 channels of the device. The signal is low pass filtered to below 150 Hz. 50 Hz line-frequency noise was band-stop filtered.

In vivo, electrophysiological measurements recorded from the brain surface of a single, acutely prepared, and deeply anesthetized mouse were performed to confirm that the original electrical recording function of the UCNP-complemented device is preserved. The device was laid on the brain surface with the *dura* intact, covering most of the left hemisphere, including visual and somatosensory areas. To help keep the device in place and provide close contact with the brain and recording sites during the measurements, the craniotomy with the device inside was partly and temporarily covered with a coverslip. Ketamine–xylazine anesthesia evokes widely described, characteristic electrophysiological patterns [52,53,54,55,56], which we used to validate our recordings. A 10 s-long sample section of a recording is presented in Figure 10, displaying the ketamine-induced slow oscillations on the recording sites. The traces were low-pass filtered below 150 Hz, and the 50 Hz line-frequency noise was filtered out using a band-stop filter. Impedances of the recording sites during the in vivo measurements were 65.38 kΩ on average (24.2–188 kΩ) at 1 kHz. These were in agreement with the EIS recordings regarding the order of magnitude. The in vivo measurements displaying local field potential oscillations with excellent quality provided ample support to the unaltered electrophysiological capability of the UCNP-complemented devices.

Biocompatibility testing of the UCNP-complemented ECoG system in vitro or in vivo was not part of this project; however, before long-term implantation, it will be crucial to examine the structure from this point of view as well. Previous inspections of silica-coated NaYF_4_ UCNPs in contact with cells showed minimal to mild cytotoxicity [57,58,59], which is a promising sign in our case as well.

## 4. Conclusions

In this work, we investigated how upconverting nanoparticles and a drug modeling dye can enhance the functionality of an electrocorticography electrode array that was implantable under in vivo conditions. We showed by various measurement methods (contact angle, FTIR) that the necessary surface modification of the UCNPs and the ECoGs was developed, i.e., hydroxyl, triethoxysilane, and BCN-NHS coating was formed on their surfaces. By these chemical layers, the two subsystems could stably link to each other, which was confirmed by mechanical (agar gel) testing combined with SEM imaging. Electrochemical impedance spectroscopy confirmed that there was no significant change in the electrical properties of the ECoGs after the nanoparticle attachment, allowing the device to be implanted later under electrophysiological conditions. The photo uncaging of a fluorescent probe from the UCNP surface was successfully demonstrated using fluorometric measurements. Two-photon imaging suggests that the illumination of the UCNPs under in vitro conditions also results in the conversion of near-infrared light to the blue wavelength regime, which means that the background fluorescence generated by the ECoG substrate material (PDMS) increases due to the detached dye’s emission. Finally, in vivo electrophysiology confirmed that the presence of the anchored and modified upconverting nanoparticles does not hinder the recording capabilities of the modified ECoG implants. In view of these findings, we believe that our model drug-releasing scheme implemented on a silicone-based brain–machine interface will open new possibilities to complement the surface of other implantables with upconverting nanoparticle-mediated functionalities.

## Figures and Tables

**Table 1:** Properties of each substrate region on three modified devices.

Region	Device 1		Device 2		Device 3		Average	
	Coverage [%]	Number of Objects	Coverage [%]	Number of Objects	Coverage [%]	Number of Objects	Coverage [%]	Number of Objects
1	53.76	5425	56.59	5963	51.40	4842	53.92	5410
2	56.52	5643	39.75	3905	37.23	4153	44.50	4567
3	55.17	5342	58.81	5423	38.42	3787	50.80	4851
4	57.61	5036	59.95	5641	45.34	4706	54.30	5128
5	59.78	5145	59.45	5750	43.87	4145	54.37	5013
6	59.48	5378	57.62	5352	39.45	3792	52.18	4841
7	57.21	5346	58.89	5241	46.80	4942	54.30	5176
8	59.21	5193	59.44	5175	44.78	5454	54.48	5274
9	59.89	5137	58.84	5765	39.64	4601	52.79	5168

## Data Availability

The datasets generated and/or analyzed during the current study are available from the corresponding author upon reasonable request.

## Supplementary Materials

The following supporting information can be downloaded at: https://www.mdpi.com/article/10.3390/s24247987/s1, Figure S1: (a) Layout of the CorTec micro 8 hexagonal ECoG array [60], (b) Simplified EIS measurement setup; Figure S2: UCNP structure (a) Multilayer, core-shell structure of our UCNPs [61] (b) Visible multilayers under SEM (scalebar indicates 200 nm).
