# Supplementary material for: Optically Controlled Drug Delivery Through Microscale Brain–Machine Interfaces Using Integrated Upconverting Nanoparticles
Source: Sensors (Basel). 2024 Dec 14;24(24):7987. doi: 10.3390/s24247987 (PMC11680031; doi:10.3390/s24247987)
Supplement: Supplementary file 1 [file sensors-24-07987-s001.zip › sensors-3358363-supplementary.pdf]

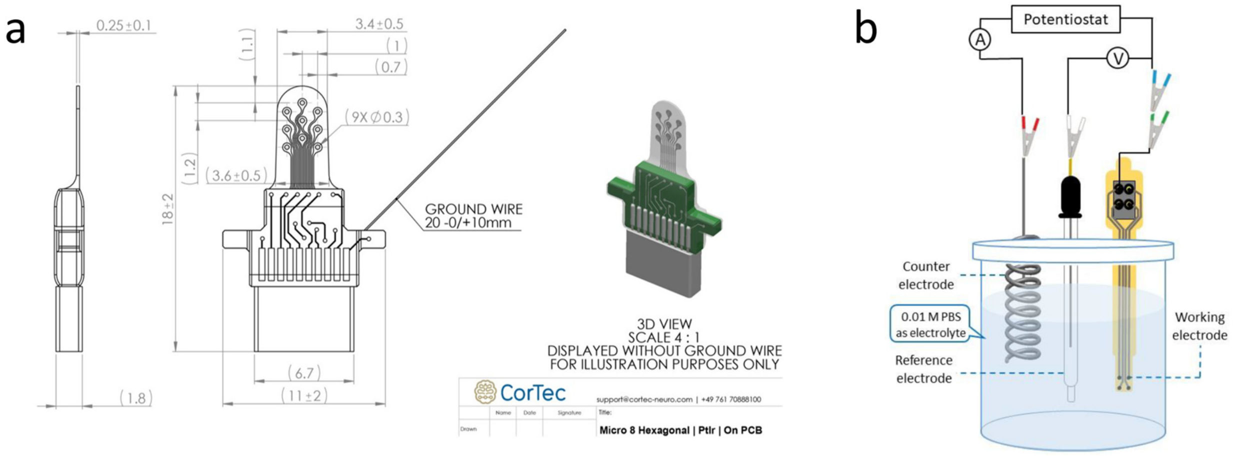

Figure S1: (a) Layout of the CorTec micro 8 hexagonal ECoG array [60], (b) Simplified EIS measurement setup

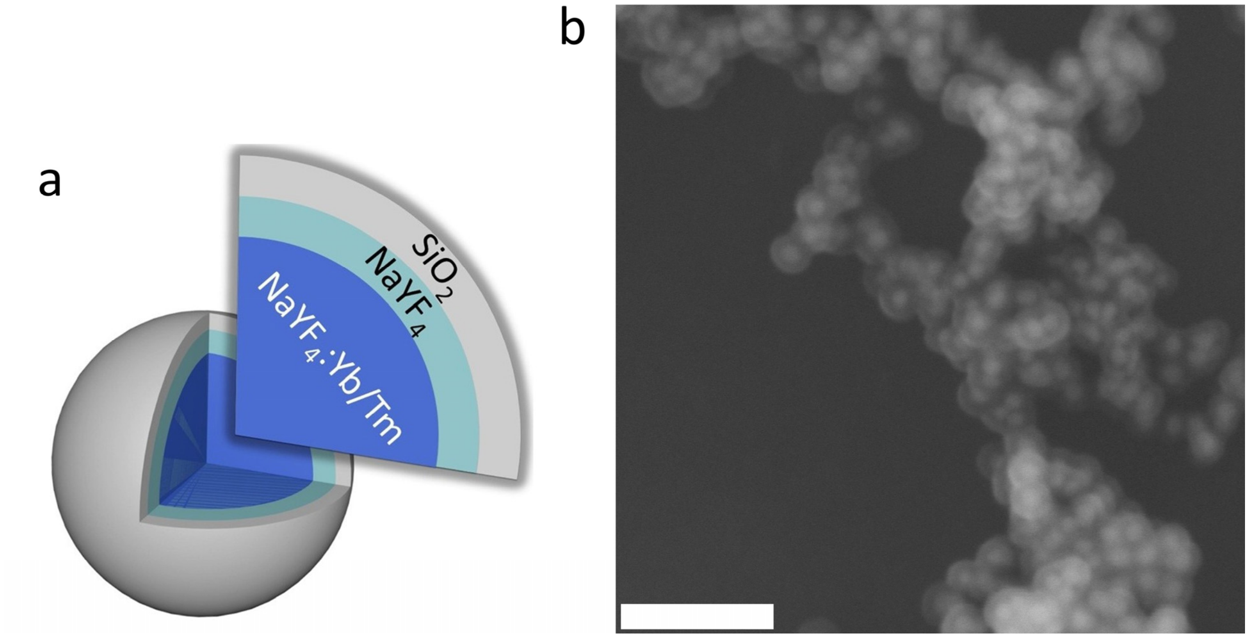

Figure S2: UCNP structure (a) Multilayer, core-shell structure of our UCNPs [61] (b) Visible multilayers under SEM (scalebar indicates 200 nm)

60. Cortec AirRay Grid Micro 8 Hexagonal ECoG Electrode. Available online: https://www.cortec-neuro.com/products-and-services/electrodes/grid-and-strip/ (accessed on 29 August 2024).

61. Chen, S.; Weitemier, A.Z.; Zeng, X.; He, L.; Wang, X.; Tao, Y.; Huang, A.J.Y.; Hashimotodani, Y.; Kano, M.; Iwasaki, H.; et al. Near-infrared deep brain stimulation via upconversion nanoparticle–mediated optogenetics. *Science* **2018**, *359*, 679–684. https://doi.org/10.1126/science.aaq1144.
